# Mechanisms of neuroinflammation in hydrocephalus after intraventricular hemorrhage: a review

**DOI:** 10.1186/s12987-022-00324-0

**Published:** 2022-04-01

**Authors:** Katherine G. Holste, Fan Xia, Fenghui Ye, Richard F. Keep, Guohua Xi

**Affiliations:** 1 Department of Neurosurgery, University of Michigan, 3470 Taubman Center, 1500 E. Medical Center Dr, Ann Arbor, MI 48109-5338 USA; 2 Department of Neurosurgery, West China Hospital, Sichuan University, Chengdu, China; 3 5018 BSRB, 109 Zina Pitcher Place, Ann Arbor, MI 48109-2200 USA

**Keywords:** Intraventricular hemorrhage, Posthemorrhagic hydrocephalus, Neuroinflammation, Complement, Microglia, Macrophages

## Abstract

Intraventricular hemorrhage (IVH) is a significant cause of morbidity and mortality in both neonatal and adult populations. IVH not only causes immediate damage to surrounding structures by way of mass effect and elevated intracranial pressure; the subsequent inflammation causes additional brain injury and edema. Of those neonates who experience severe IVH, 25–30% will go on to develop post-hemorrhagic hydrocephalus (PHH). PHH places neonates and adults at risk for white matter injury, seizures, and death. Unfortunately, the molecular determinants of PHH are not well understood. Within the past decade an emphasis has been placed on neuroinflammation in IVH and PHH. More information has come to light regarding inflammation-induced fibrosis and cerebrospinal fluid hypersecretion in response to IVH. The aim of this review is to discuss the role of neuroinflammation involving clot-derived neuroinflammatory factors including hemoglobin/iron, peroxiredoxin-2 and thrombin, as well as macrophages/microglia, cytokines and complement in the development of PHH. Understanding the mechanisms of neuroinflammation after IVH may highlight potential novel therapeutic targets for PHH.

## Introduction

Intraventricular hemorrhage (IVH) is a significant cause of morbidity and mortality after germinal matrix hemorrhage (GMH) in neonates and intracranial hemorrhage in adults. Infants with very low birth weight (< 1,500 g) are particularly at risk for GMH-IVH, with 22% of this population going on to develop GMH-IVH [1]. Of those neonates who develop high grade IVH, about 25–30% develop post-hemorrhagic hydrocephalus (PHH) [1, 2]. Left untreated, hydrocephalus can contribute to white matter injury, seizures, cognitive impairment and even death [3].

In adults, IVH occurs due to intraventricular extension of subarachnoid hemorrhage (SAH) or intracerebral hemorrhage (ICH) or occasionally choroid plexus hemorrhage [4, 5]. PHH develops in up to two thirds of patients with IVH [6]. IVH is an independent predictor of worse clinical outcome and increased mortality after ICH [7–9]. Two large, randomized control trials of adult patients with ICH demonstrated that IVH with or without hydrocephalus was associated with poor functional outcome. Patients with IVH and PHH had even worse functional outcomes [6, 8, 10].

In both adults and children, PHH requires cerebrospinal fluid (CSF) diversion either through a temporary external drain, access reservoir, permanently implanted shunt device, or endoscopic third ventriculostomy. These devices have a high rate of failure, quoted to be up to 40% within one year of surgery [11, 12], as well as risk of infection which can lead to potentially lethal ventriculitis or meningitis [13, 14]. This is in addition to the long-term psychosocial burden associated with shunt devices affecting quality of life in both children and adults [15]. Though prior studies have tried ventricular drainage, irrigation and fibrinolytic therapy in pediatric patients with GMH-IVH, they were unsuccessful in preventing the need for a shunt [16]. In adults with IVH, the data are more mixed with some studies showing a reduced need for shunting after administration of intraventricular fibrinolytics after IVH [17–20]. In the case of GMH-IVH, prevention is crucial, a complete discussion of which is outside the scope of this review [21, 22].

The aim of this focused review is to discuss the advances in understanding of mechanisms of neuroinflammation within the ventricle, choroid plexus, and periventricular tissue in PHH after IVH in both neonates and adults. Neuroinflammation was defined as the inflammatory response within the brain produced by resident and peripheral circulating inflammatory cells and mediated by reactive oxygen species, cytokines/chemokines and other messenger molecules [23, 24]. Inflammation within the leptomeninges and arachnoid villi is an important topic, but outside the scope of this focused review [25, 26]. The most relevant studies on this topic were identified through MEDLINE (accessed by PubMed on 11/19/2021) using the following terms: ‘hydrocephalus’ and ‘intraventricular hemorrhage’, ‘subarachnoid hemorrhage’ and ‘intracranial hemorrhage’ as well as ‘posthemorrhagic hydrocephalus’. The reference lists for each article were reviewed to search for additional relevant studies. Both preclinical and clinical studies were included if they reported mechanisms of PHH after IVH, specifically the role of inflammation.

### Intraventricular hemorrhage etiology

In the pre-term neonatal population, IVH is most commonly due to GMH. The germinal matrix is an area of rapid cell proliferation, located between the wall of the lateral ventricle and the caudate nucleus and is present until approximately 34 weeks gestation [27]. Cells from the germinal matrix later develop into neuronal and glial cells. Given its rapid proliferation, this is a highly vascularized structure full of immature vessels [28, 29]. These vessels lack not only the supportive connective tissue seen in adult vessels, but also a reduced number of pericytes which are important for maintaining the blood brain barrier [27, 30, 31]. These characteristics of fragile germinal matrix vessels make them extraordinarily prone to rupture. The necessary inciting event for rupture is unknown but is hypothesized to involve rapid changes in blood flow during the neonatal period. In contrast, the germinal matrix is no longer present in the term neonate where IVH is thought to result from choroid plexus hemorrhage [32–34]. GMH is graded based on the extent of hemorrhage and ventriculomegaly. Severe GMH-IVH, grade III or IV, is defined by the presence of ventricular dilation and intraparenchymal hemorrhage respectively. A more severe GMH-IVH grade is associated with increased incidence of PHH and greater morbidity and mortality within these neonates [35–37].

In preterm infants, GMH-IVH contributes to white matter injury through mass effect on the corpus callosum and corona radiata, elevated intracranial pressures, reduced cerebral blood flow as well as activation of the subsequent inflammatory cascade. GMH-IVH and PHH are not the sole causes of white matter injury in the preterm neonate: hypoxic-ischemic events can contribute significantly to white matter injury in this population [38, 39].

Using animal models to determine neurodevelopmental outcomes in preterm infants can be challenging. Researchers often use post-natal day 1–10 rats to model third trimester human fetuses based on prior research comparing brain growth and protein expression [40, 41]. Newer models of brain maturation in a wide variety of mammals have contributed to understanding neurodevelopmental correlates with humans [42]. Based on these findings, limitations of the animal models are coming to light. Altricial (early) compared to precocial (late) timing of birth in relation to brain maturation has significant effects on the timing and speed of neurogenesis. For example, rodents, though beneficial for their rapid development, are altricial whereas humans are more ambiguous and therefore their brain maturation speeds are likely very different [42].

In adults, IVH is often secondary to ICH, SAH, other vascular malformations or trauma. IVH occurs in 42–49% of patients with ICH and about 15% of patients with SAH [8]. Older age, hypertension, and increased volume of ICH are associated with IVH development [10]. There is a continuous relationship between the volume of IVH and mortality [8]. Grading IVH is much more cumbersome in adults than in neonates; most scores incorporate IVH as a component of ICH or SAH outcome scores or attempt to estimate IVH volume [43]. Graeb and colleagues published a scoring system for severity of IVH based on calculating the amount of blood within the ventricular system. A greater burden of ventricular blood was associated with worse prognosis: in their study 90% of severe IVH patients died [44]. Though the etiology of IVH in neonates and adults are very different, both populations are at risk of developing PHH and the treatment of PHH remains the same.

### Theories of PHH etiology

**Fig. 1:**
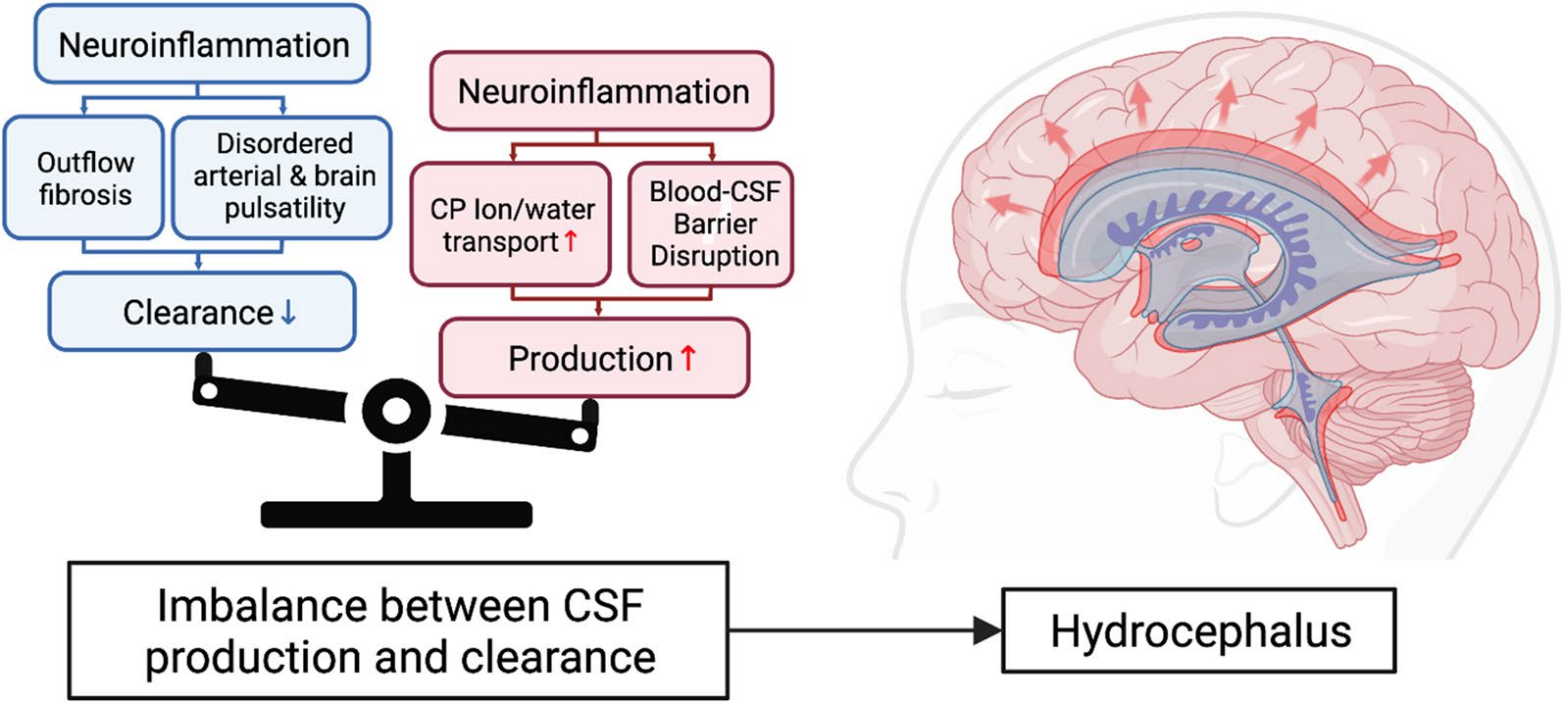
The imbalance of CSF production/drainage induces hydrocephalus. AfterIVH, CSF drainage is impaired due to inflammation at the leptomeninges and likely causes changes in brain pulsatility.. CSF hypersecretion mainly occurs at the choroid plexus with possible blood-CSF barrier disruption and AQPs upregulation. IVH, intraventricular hemorrhage; CSF, cerebrospinal fluid; AQPs, aquaporins

The bulk flow model of hydrocephalus dates to Dandy and Blackfan’s original research on hydrocephalus (see historical review) [45]. In that study they reproduced hydrocephalus by plugging the cerebral aqueduct in dogs with a sponge leading to the hypothesis that hydrocephalus results as an imbalance between CSF production and clearance (Fig. 1). In their original classification system, they defined non-communicating hydrocephalus as hydrocephalus due to an overt obstruction within the ventricular system, such as a mass lesion or aqueductal stenosis. Conversely, communicating hydrocephalus was defined as ventriculomegaly without the presence of an obstructive lesion, resulting in enlargement in all four ventricles [46].

For many years, the primary focus of PHH research has been on reduced CSF absorption, in particular, due to obstruction of the arachnoid villi and granulations from blood components [47, 48]. Arachnoid granulations as the primary site of CSF absorption was proposed by Dandy in 1919 using his experimental model of hydrocephalus using India-ink in animal models [49]. However, there are a number of issues with this model; preterm infants, who make up a majority of neonates with PHH, do not have arachnoid granulations until they are at term and animals such as rodents do not have arachnoid granulations and have very few arachnoid villi [50, 51]. Extensive studies have shown that there are multiple sites of CSF absorption along cranial (including olfactory and optic) nerves and spinal nerves to the lymph system and a newly described meningeal lymph system [52, 53]. The arachnoid granulations/villi may be a ‘safety valve’ when CSF pressure is elevated. There is a growing body of evidence that inflammation plays a major role in PHH, especially in neonatal PHH, impacting CSFclearance (Fig. 1).

Along the same lines of reduction in CSF absorption, damage or fibrosis of the ependyma may contribute to PHH. The ependyma is an epithelial layer with motile cilia, primary cilia and microvilli [54]. The cilia on the cell surface are thought to direct CSF flow and genetic models of ciliary dysfunction has been associated with hydrocephalus [26, 55–57]. Damage to the ependyma directly through blood products or increased intracranial pressures can lead to discontinuity of the surface, gliosis and scarring. Postmortem pathological studies of infants with IVH and PHH found reduction in ciliated ependymal cells and defective adhesion between cells associated with altered CSF dynamics [58, 59]. In animal models, ependymal and subependymal injury are seen after IVH and are worse in those that develop PHH [60, 61]. Microstructural changes to the ependymal motile cilia were seen on electron microscopy in one neonatal rat model of communicating hydrocephalus [54, 62]. Additionally in a genetically altered mouse model, programmed denudation of the ependyma preceded hydrocephalus development [63]. After IVH, in one rat model, increased inflammatory markers were seen in the damaged ependymal layer as well as increased permeability to proteins such as IgG [64]. As ependymal cells have questionable ability to regenerate, ependymal injury may have long-lasting effects [65–67]. The exact contribution of ependymal/ciliary damage to PHH, if any, is unknown.

A relatively newly described etiology of communicating hydrocephalus involves aberrant brain pulsations. Increased intracranial pressures lead to reduced brain compliance, as the brain is in a fixed container, which changes the cardiac-induced pulsatility of the brain. A thorough review of these concepts are outside of the scope of this article and can be found in this recent review [68]. In hydrocephalus, increased intracranial pressures and tissue changes, such as cerebral edema, can change the compliance of the brain and therefore its pulsatility. Changes in brain pulsatility alter the normal flow dynamics of CSF and can lead aberrant CSF drainage [69–71].

The majority of CSF production occurs at the choroid plexus with a fraction produced via flow across the blood–brain barrier and ventricular ependyma [72]. The choroid plexus is a very vascular organ present within all four ventricles within the brain and forms the blood-CSF barrier. The choroid plexus epithelium has a polarized distribution of ion transporters on the apical and basolateral membranes and a water channel, aquaporin1, that are only present on the apical membrane. This polarity results in a vectoral movement of ions and water from blood to CSF forming the basis for CSF secretion. That secretion can be regulated by systemic signals, but the exact mechanisms are not well understood [73]. There is growing evidence that alterations in CSF production, including those due to neuroinflammation, contributes to hydrocephalus [74].

How CSF secretion and underlying mechanisms are altered during development are a relatively understudied area. This has been highlighted recently by the study of Xu et al. examining the role of the sodium potassium chloride cotransporter, NKCC1, at the apical membrane of the choroid plexus epithelium [75]. They found evidence early in mouse development that this transporter is involved in clearing ions and water from the CSF towards blood. These findings also provide evidence that the choroid plexus not only plays a role in CSF production, but also has some absorptive capabilities. This contrasts with the adult rat where Karimy et al. found that NKCC1 transported ions and water from the epithelium into CSF contributing to CSF production [74]. Developmental changes in the choroid plexus may play a significantly different role in hydrocephalus between pediatric and adult populations.

### Inciting factors of inflammation after IVH

**Fig. 2:**
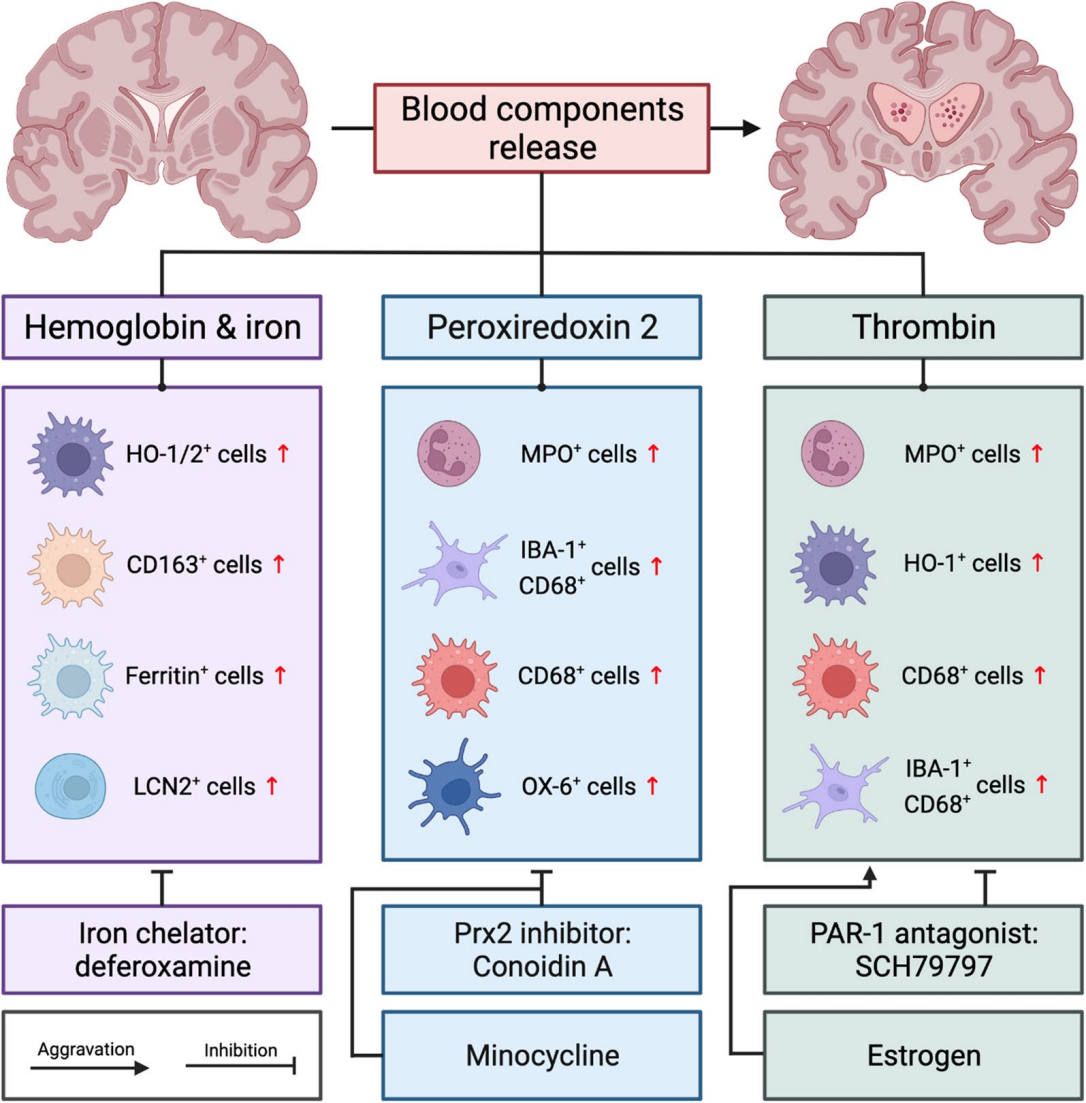
Blood components release triggers neuroinflammation and contributes to subsequent hydrocephalus. Hemoglobin and its primary metabolite iron, peroxiredoxin 2, and thrombin can induce hydrocephalus via pro-inflammatory activated macrophages (red CD68 + cells)/resident microglia (blue CD68 + cells), epiplexus cells (OX6 +), and granulocytes (MPO +).. Blocking blood components’ activity curtails neuroinflammation and alleviates post-hemorrhagic hydrocephalus. Estrogen can exacerbate thrombin-mediated brain injury and aggravate hydrocephalus. HO-1/2, heme oxygenase; LCN2, lipocalin-2; MPO, myeloperoxidase; IBA-1, ionized calcium binding adaptor molecule 1; OX-6, major histocompatibility complex II expressed by epiplexus cells; Prx2, peroxiredoxin 2; PAR-1, protease-activated receptor-1; SCH79797, 3-N-cyclopropyl-7-[(4-propan-2-ylphenyl)methyl]pyrrolo[3,2-f]quinazoline-1,3-diamine;dihydrochloride

After IVH, erythrocytes, a major component of blood, are released into the ventricular system. When these cells lyse, their potentially neurotoxic components are released into the CSF. These components include hemoglobin (Hb), iron, peroxiredoxin-2 (Prx2), and carbonic anhydrase-1 [76–79]. These components along with plasma proteins, including thrombin, can cause inflammation contributing to secondary brain injury and possibly PHH, as seen in ICH, SAH and IVH models (Fig. 2) [80–84].

#### Erythrocyte components, Hb/Iron, and Prx-2

Hb from lysed erythrocytes is a potent activator of inflammation. Free Hb dissociates into its alpha and beta dimers which are then scavenged by haptoglobin (Hp). Although normal CSF Hp levels are very low some will enter the brain with the initial hemorrhage [85, 86]. In addition, at least in ICH, there is evidence of increased brain Hp expression [85]. The Hb-Hp heterodimers are endocytosed via CD163, a receptor on monocytes, macrophages and even neurons [87–89]. Once inside cells, heme moieties are degraded by either heme-oxygenase (HO)-1 in macrophages/microglia or HO-2 in neurons to form biliverdin, carbon monoxide, and ferrous iron [90]. This is seen in vivo as intraventricular injection of lysed erythrocytes causes increased HO-1 activity [91]. The iron released by Hb breakdown can cause oxidative damage to nearby tissues through Fenton reactions. This has been demonstrated in animal models of SAH, IVH and ICH [92–94]. Iron accumulation was correlated with lateral ventricle dilation, cerebral edema, neuronal degeneration in the basal ganglia and decreased long term motor function in a rat model of IVH [95]. Additionally, in another study, structural damage was seen in the nearby choroid plexus and hippocampus after IVH via iron induced JNK signaling pathways [87]. Upregulation of other pro-inflammatory iron handling proteins, such as Lipocalin-2 (LCN2), occur after ICH. Elimination of LCN2, through the use of knockout mice, demonstrated less microglial activation after ICH [96]. Hb and iron mediated brain injury after hemorrhage is supported widely by the literature [97, 98].

Hb and iron appear also to be involved in the mechanism of PHH development in adult and neonatal animal models. Injection of whole blood in an adult rat IVH model caused PHH in 80% of rats at days 7 and 28 after ictus. The effects were similar when injecting iron (FeCl_3_): PHH was seen in 60% and 70% of adult rats at days 7 and 28 respectively after ictus [99]. Additionally, injection of lysed erythrocytes or elemental iron caused rapid ventricular enlargement and death in another adult rat IVH study [35]. These results are not isolated to adult IVH: in a neonatal rat model of GMH-IVH, intraventricular injection of Hb and FeCl_3_ resulted in significantly larger ventricular size as compared to artificial CSF. In contrast, injection of protoporphyrin IX, the iron-less precursor of heme, did not increase ventricular size compared to artificial CSF [100]. That study demonstrated that iron was the necessary component of Hb to induce ventriculomegaly. Histologically, free iron and iron-handling proteins like HO-1 and ferritin were increased with increased ventricular size [100, 101]. Deferoxamine, an iron chelator, significantly reduced the risk of hydrocephalus to 20% of rats in whole blood injections and 10% of the iron injection group in an adult IVH model [99]. Functionally, the addition of deferoxamine attenuated the prolonged maze escape time seen with hydrocephalic rats 28 days after hemorrhage [99].

In addition to brain injury through reactive oxygen species, iron may play an additional role in fibrosis of the ventricles and CSF hypersecretion. Intraventricular injection of iron was found to cause an increased level of Wnt mRNA and protein within the brain at 7 and 28 days after IVH. Wnt signaling is important in fibrosis formation, as seen in other conditions such as liver and pulmonary fibrosis [102, 103]. The effect on Wnt was attenuated with deferoxamine leading the authors to conclude that iron may play a role in subarachnoid fibrosis, and scarring, leading to PHH [99]. Fibrosis, measured using alpha-smooth muscle actin as a marker, was also seen within organized hematomas in aged rats after IVH. These organized hematomas presented with larger lesions as seen on MRIs and were not present in young rats with IVH [104].

Aquaporins (AQP) are channels that facilitate water movement across cell membranes. They are found in the choroid plexus (mainly AQP1) and along the ependyma (AQP4) and are thought to be major contributors to CSF homeostasis [105]. Qing and colleagues found an upregulation of AQP4 expression in the perihematomal region 3 days after ICH which persisted until day 14 and was attenuated with administration of deferoxamine [106]. They concluded that iron played a role in ICH induced brain edema through AQP4 upregulation. Recently, in a rabbit pup model of GMH-IVH in which spontaneous hemorrhage was induced, AQP5 mRNA and protein expression was upregulated along the apical border of the choroid plexus at 24 and 72 h after hemorrhage [107]. It will be important to examine the role of iron in AQP5 upregulation after IVH.

The importance of Hb and iron in PHH is not restricted to animal models. A small study of infants with PHH found 75% had non-protein bound ferrous iron in their CSF [108]. Additionally, a recent prospective multicenter study examined infants with GMH-IVH and PHH who underwent temporary or permanent CSF diversion. Their CSF was analyzed for components of the iron metabolism pathway. There was a reduction in CSF hemoglobin, iron, total bilirubin and ferritin as well as an increase in hemopexin, a heme scavenger, levels between time of temporary and permanent CSF diversion. Neonates with higher CSF ferritin levels at the time of permanent CSF diversion were more likely to have larger ventricular size and those with a higher concentration of hemopexin were more likely to have a smaller ventricular size. A larger reduction in ferritin levels between temporary and permanent CSF diversion was associated with improved functional outcomes [3]. The authors proposed that components of the iron metabolism pathway may influence development of PHH and could be prognostic of functional outcome.

Hemoglobin is not the only potential inflammatory factor inside erythrocytes. Peroxiredoxin-2 (Prx2) is the 3rd most prevalent protein in red blood cells and upon release into the extracellular environment it is a potent proinflammatory factor [109]. Prx2 protein levels were found to be elevated in the periventricular zone 1 h after IVH [110]. Intraventricular administration of Prx2 caused hydrocephalus as well as ependymal damage and macrophage activation [76, 110]. Co-injection of a PRX2 inhibitor demonstrated a significant reduction in ventricular size, ependymal damage and inflammatory cell accumulation [110].The effects of Prx2 in relation to PHH merit further investigation.

#### Thrombin

After hemorrhage, the coagulation cascade is promptly activated. Thrombin, or factor IIa, is a serine protease that induces blood clotting by cleaving fibrinogen into fibrin. Thrombin itself is a potent activator of inflammation either by ischemia (from thrombus generation), directly through protease activated receptors (PAR), or indirectly through leukocyte recruitment [111]. Experimentally, injection of heparinized blood into the ventricles reduced the risk of hydrocephalus as compared to whole blood indicating that a component of the coagulation cascade may play a role in PHH development [112, 113]. The use of heparinized blood injection may be an important confounder in ICH/IVH research [114–116]. Intraventricular injection of thrombin alone caused significant hydrocephalus, ventricular wall damage and blood–brain barrier disruption [112, 117]. Indeed, rats injected with thrombin alone had ventricular volumes double that of controls [118]. This is not limited to animal studies, thrombin concentration and activity in the CSF of ICH patients was elevated compared to controls. Elevated thrombin concentration and activity were associated with poor functional outcome at 6 weeks and 6 months [119]. In the ventricles, thrombin can interact with different PAR family members. PAR-1 is a G protein-coupled receptor activated by thrombin and was seen to induce ependymal wall damage and hydrocephalus [113]. Using SCH79797, a PAR-1 antagonist, reduced cerebral edema and blood brain barrier disruption after ICH [113, 120] as well as reduced ventricular dilation after IVH [112]. Thrombin also appears to play a role in blood-CSF barrier disruption. In a rat model of IVH, intraventricular injection of thrombin was seen to downregulate VE-cadherin within the choroid plexus, an essential component of the blood-CSF barrier [112]. Downregulation of VE-cadherin contributed to increased vessel permeability which may contribute to hydrocephalus development. Additionally, thrombin increased the number of surrounding activated macrophages and microglia at 24 h [121]. This increase in macrophages and microglia could be through the same blood-CSF barrier disruption mechanism allowing increased macrophage migration or by acting like a chemoattractant. A recent study revealed that neutrophils were heavily recruited in choroid plexus as well as periventricular zone in a thrombin induced hydrocephalus model, giving evidence that neutrophils are deeply involved in thrombin-mediated injury [83]. Further study and intervention on neutrophils in the thrombin model are warranted.

### Components of the inflammatory response

#### Macrophage activation and migration

After hemorrhage, microglia, the resident immune cells within the brain, and infiltrating macrophages are activated. In the Clot Lysis: Evaluating Accelerated Resolution of Intraventricular Hemorrhage (CLEAR) III trial, leukocyte levels peaked at 2–3 days after IVH, lasted for 3–5 days and roughly correlated with the volume of IVH in adult humans [122]. In addition to phagocytosis of erythrocytes and their debris, resident microglia and recruited macrophages can secrete pro-inflammatory cytokines, extracellular proteases, and oxidative species [124]. The pro-inflammatory, M1 activated phagocytes are associated with increased brain injury after hemorrhage. Utilization of minocycline to dampen microglia activation attenuated inflammation after hemorrhage as seen on histology [125]. Conversely, the M2 activated phagocytes secrete anti-inflammatory cytokines, may help in clearance of cell debris as well as promote healing [126, 127]. As such, understanding how to dampen the pro-inflammatory response and improve the anti-inflammatory response are potential targets for attenuating brain injury and edema after IVH.

Enhancing erythrocyte clearance by manipulating endogenous macrophages and microglia has been an area of intense research focus, particularly in ICH [80, 128]. One approach focuses on CD47, a receptor present on the membranes of erythrocytes that acts as a “do not eat me” signal to macrophages. Animals that are genetically deficient in CD47 or those administered CD47 inhibiting antibodies have accelerated hematoma clearance, reduction in brain edema, reduced neuronal death and improved functional outcomes in ICH models [129–131]. In an adult rat IVH model, co-injection of a CD47 blocking antibody resulted in significantly smaller ventricular volumes 3 days after ictus than those co-injected with immunoglobulin. Co-injection of the CD47 blocking antibody accelerated hematoma clearance and reduced hemolysis visualized within the hematoma. There were an increased number of activated macrophages within the hematoma in this group. When monocytes and macrophages were depleted with clodronate liposomes, there was worse and progressive ventriculomegaly [131]. Similarly, in a rat ICH model, depletion of phagocytes reduced hematoma clearance, worsened brain edema, neuronal loss and behavioral deficits [129]. This is not unique to animal studies. In adult patients with IVH, the extent of hematoma clearance was significantly associated with CSF leukocyte count, indicating a more robust inflammatory response aided in hematoma clearance [122, 123, 132].

Promotion of M2 microglia/macrophage differentiation is another potential target to enhance hematoma clearance. CD36 is scavenger receptor and mediates erythrophagocytosis. Upregulation of CD36 has been seen to promote hematoma resolution after hemorrhagic stroke [133] and stimulation promotes M2 microglia differentiation [134]. A neonatal GMH-IVH rat model where CD36 was depleted by siRNA, showed a reduction in hematoma clearance [135]. Peroxisome proliferator-activated receptor gamma (PPAR-γ) is an upregulator of CD36. Using the same neonatal rat GMH-IVH model, stimulation of PPAR-γ enhanced hematoma resolution, reduced white matter loss, ventricular dilation, and improved functional outcomes. PPAR-γ stimulation increased the number of CD206 positive, or M2 activated microglia in the perihematomal region [135]. Not only did enhanced hematoma clearance improve ventriculomegaly, brain edema and functional outcomes, but reduced hematoma clearance worsened these outcomes which suggests that improving hematoma clearance could be a therapeutic target for IVH and PHH.

There may be both a temporal and regional importance in macrophage activation. Researchers have been interested in learning the mechanism of preferential white matter injury after IVH and in PHH. Macrophage activation may play a temporal and regional role in this. Increased presence of microglia/macrophages within the periventricular white matter has been observed after IVH in a number of animal models [136–140]. In a rat pup model of GMH-IVH, there was a significant increase in number of macrophages within the periventricular white mater at 3 and 24 h after IVH. A similar increase at 3 and 24 h was not present in the gray matter. Interestingly, there were also increases in CD68 positive cells, macrophages/microglia with increased lysosomal activity, in the white matter and gray matter at 3 and 24 h. A similar effect was seen in the contralateral hemisphere as well [82].Temporal and regional activation of macrophages may be important in understanding white matter injury after IVH. In subsequent analyses of the CLEAR III trial, researchers found neutrophils were the first circulating inflammatory cells in the CSF of adults after IVH. Infiltration of other leukocyte populations, such as lymphocytes and monocytes were found to spike later, on day 2 after IVH indicating temporal deviations in cell types within the CSF [123, 132]. The temporality and the role of different leukocytes in the ventricle and CSF after IVH are areas in need of further study.

**Fig. 3:**
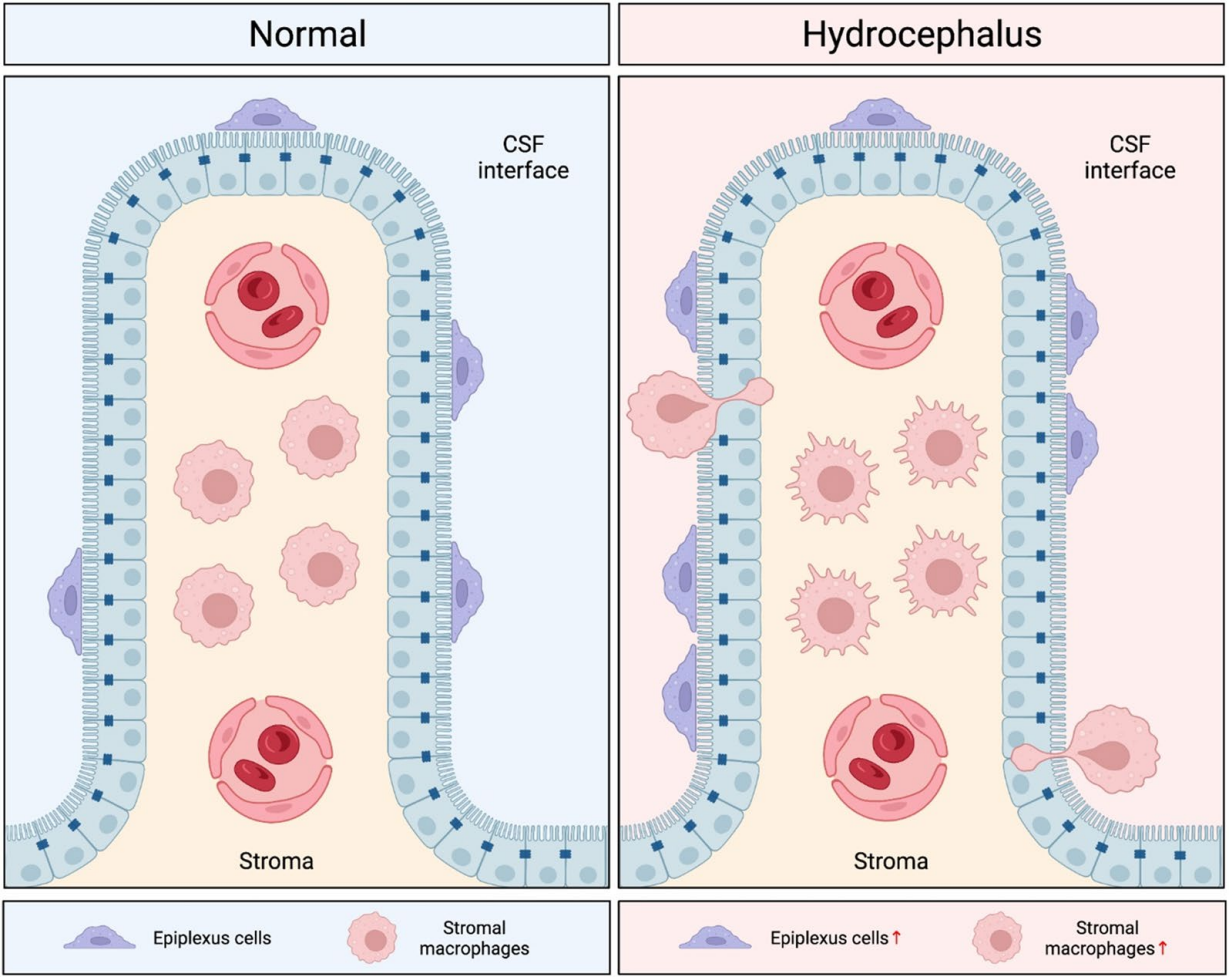
Kolmer’s epiplexus cells and stromal macrophages hold a crucial role in post-hemorrhagic hydrocephalus genesis. Activation and upregulation of epiplexus cells and stromal macrophages contribute to neuroinflammation and hydrocephalus. Moreover, stromal resident macrophages tend to move to the choroid plexus apical surface after injury stimulation

Understanding the role of choroid plexus epiplexus macrophages (Kolmer cells) in PHH development is a new area of research (Fig. 3). Epiplexus macrophages are cells on the ventricular (apical) side of the choroid plexus and are thought to act as antigen presenting cells [141]. One study injected Prx2, a component of erythrocytes which is proinflammatory when released extracellularly, into the ventricles of adult rats. After 24 h, the number of epiplexus cells increased and their morphology changed with larger soma size. Additionally, injection of Prx2 caused hydrocephalus and ventricular wall damage at 24 h which was attenuated with depletion of the epiplexus cells [76]. In a rat SAH model, rats with PHH had greater number of activated epiplexus cells and larger soma size than rats without PHH [121]. The role of epiplexus cells after IVH and in PHH is poorly understood and requires further exploration.

While resident choroid plexus macrophages are one source of leukocytes impacted by IVH, leukocytes may also infiltrate into the CSF and brain across the choroid plexus and meninges (which together form the blood-CSF barrier) as well as the blood–brain barrier. Given the proximity of the choroid plexus to an intraventricular hematoma, the choroid plexus may be an important site of infiltration in IVH and there is evidence that the choroid plexus is an important early site infiltration in experimental autoimmune encephalomyelitis (EAE; a model of multiple sclerosis) [142]. However, the relative importance of the blood-CSF and blood–brain barriers as sites of entry in IVH is still uncertain.

Leukocyte migration across barrier tissues such as the blood-CSF and blood–brain barriers involve chemokines and cytokines that direct migration but also the expression of adhesion molecules on the barrier tissue, such as intercellular adhesion molecule-1 (ICAM-1) and vascular cell adhesion molecule-1 (VCAM-1). The choroid plexus epithelium normally expresses ICAM-1 and VCAM-1 and the latter is increased in EAE. Activation of toll-like receptor 2 (TLR-2) increases choroid plexus ICAM-1 mRNA expression 60-fold [143] indicating the marked effects that inflammation can have on adhesion molecule expression at the choroid plexus. Recently, Erdei et al. found that CSF ICAM-1 and VCAM-1 levels closely correlate with CSF heme levels [144]. There is a need for studies examining adhesion molecule expression at the choroid plexus and the periventricular zone in IVH [145]. Targeting adhesion molecules may be a method of manipulating the entry of different leukocyte subsets in ICH.

#### Cytokines, chemokines and transcription factors

Inflammatory cytokines may also play a role in PHH development and brain injury after IVH. Each cytokine acts differently, but as a group, cytokines and chemokines increase vascular permeability, promote brain edema formation, recruit leukocytes, and may have direct neurotoxic effects [146]. Generation of these cytokines/chemokines after IVH can be seen in both in vitro and in vivo studies. Exposure of rabbit astrocyte cell cultures to methemoglobin, an oxidized form of hemoglobin, lead to a dose-dependent increase in tumor necrosis factor-α (TNF-α) mRNA and protein expression in one study [147]. Additionally, elevated levels of inflammatory cytokines can be seen in the CSF and periventricular tissue of animals after IVH [137, 138, 147, 148]. A study of rat pups injected with intraventricular Hb found significantly elevated levels of proinflammatory cytokines such as (TNF-α), chemokine ligand 1, interleukin 1β, and interleukin-6 acutely after injection. Evidence of oxidative stress within the white matter was seen at 3 and 24 h after injection in both the ipsi- and contralateral hemispheres. Those animals with IVH had a significant reduction in myelin basic protein 37 days after hemorrhage, indicating that these inflammatory reactions may be related to long term effects on the white matter tracts [82]. In humans, a recent multicenter study of adult patients with spontaneous ICH with IVH found elevated cytokine/chemokine concentrations within the CSF which correlated with hematoma volume and perihematomal edema [146]. Elevated CSF chemokine levels showed a significant association with poor clinical outcome after SAH [149]. Infants with GMH-IVH have been noted to have elevated inflammatory markers in their CSF such as TNF-α, which in one study was strongly correlated with the total heme levels in CSF [144, 147, 150–152]. These data supported the association between inflammatory response and ICH/IVH severity as well as cytokines as a potential therapeutic target.

Cytokines not only act to increase inflammation, but also promote fibrosis of the leptomeninges and arachnoid granulations as well protein deposition in the periventricular tissue, contributing to PHH. Transforming growth factor-β (TGF-β) is a small signaling protein important in controlling processes such as wound healing, maintenance of the extracellular matrix, angiogenesis and apoptosis [153]. All forms of TGF-β are secreted by activated microglia and can be induced by thrombin, but TGF-β1 is the most abundant isoform within the CNS [153, 154]. TGF-β1 mediated cellular fibrosis is found in various organs within the body such as the liver [155, 156]. TGF-β1 can also induce leptomeningeal cell proliferation [157] and was found to be elevated in periventricular tissue after SAH [153]. Multiple animal and human studies have demonstrated increased TGF-β1 expression after IVH, especially in animals that went on to develop PHH [158–162]. TGF-β1 was not just increased in the CSF, but also within arachnoid cells, [162] ependyma and subependymal layers indicating that these tissues were at risk for potential protein deposition [158]. TGF-β1 antagonists, such as decorin, reduced the risk of chronic hydrocephalus and improved behavioral outcomes in a rat acquired communicating hydrocephalus model [163, 164]. TGF-β1 mediated fibrosis may play a role in PHH development after IVH.

The role of CSF hypersecretion in PHH is an emergent field. Increase in nuclear factor kappa-light-chain-enhancer of activated B cells (NF-κB) signaling is seen after IVH and especially so in PHH [138, 148, 162]. In a seminal paper on inflammation and CSF hypersecretion, Karimy and colleagues found lateral ventricular CSF production tripled at 48 h after IVH and remained elevated at 7 days. This effect was thought to be mediated by NF-κB, a family of inducible transcription factors, which was activated within choroid plexus epithelial cells. A NF-κB inhibitor, administered intraperitoneally which crossed the blood–brain barrier, reduced CSF secretion rate and attenuated ventriculomegaly after IVH. The mechanism of CSF hypersecretion was thought to be due to increases in the activity of the sodium potassium chloride transporter (NKCC1) on the apical membrane of the choroid plexus. NF-κB signaling through a serine-threonine kinase, SPAK, results in phosphorylation migration of NKCC1 to the choroid plexus apical membrane. Administration of an NF-κB inhibitor reduced the amount of NKCC1 present and administration of a SPAK inhibitor reduced CSF secretion back to baseline after IVH [74]. The NF-κB pathway and NKCC1 are new exciting potential targets for PHH. Current systemically administered loop diuretics (furosemide, bumetanide) cannot penetrate the blood-CSF barrier sufficiently to inhibit the apically located NKCC1 at the choroid plexus.

#### Complement

The complement cascade’s role in IVH and PHH is vastly understudied. All potential mechanisms are extrapolated from ICH and SAH research. The complement cascade is part of the innate immune system and has been implicated in a number of neurologic diseases [165]. The terminal complex (C5b-9), known as the membrane attack complex (MAC), acts by creating a pore on the membranes of bacteria or damaged cells leading to cell lysis. The earlier components of the cascade, C3 and C5a, act as attractants for macrophages and opsonins to flag cells for phagocytosis [165, 166]. Prior studies have demonstrated early activation of the complement cascade after ICH. In a mouse model of ICH, C3 and C9 levels were found to be elevated in the perihematomal region 24 and 72 h after hemorrhage [167]. In humans, CSF levels of C5 were > 1400-fold increased on the first day after SAH with a gradual decline [168]. In another study of patients with SAH, increased plasma levels of C3a and C5a were associated with poor clinical outcome [169]. The nature of complement’s contribution to PHH is an area of active research.

The complement cascade, through MAC, plays a significant role in erythrolysis within the hematoma thereby potentially promoting Hb-, iron-, and Prx2-mediated cell damage and inflammation. N-acetyl-heparin is a derivative of heparin that can block the complement cascade without the anticoagulant effects of heparin. When co-injected in an animal ICH model, there was a significant reduction in erythrolysis at 24 and 72 h after ICH, reduced perihematomal iron accumulation at day 28 and a reduced number of HO-1 positive cells. Co-injection of aurin tricarboxylic acid, a MAC inhibitor, similarly reduced erythrolysis at 24 and 72 h and reduced HO-1 expression [170]. These data indicate that blocking the complement cascade could be a potential upstream target to reduce erythrolysis.

Complement also acts by attracting phagocytes toward an area of injury: a response that can be beneficial or detrimental. When C3b complexes are deposited on apoptotic or necrotic cells, phagocytosis and removal of this debris is promoted by macrophages/microglia [171]. It has been hypothesized that improved opsonization using complement complexes would facilitate hematoma clearance [172]. Activation of the early complement cascade to increase these opsonization factors could be an upstream target, but there is limited data to support this. Attracting phagocytes to an injured area does come with a price: pro-inflammatory cytokines secreted by activated phagocytes may cause additional brain injury. Complete systemic complement depletion, using cobra venom factor, reduced brain edema and toxic cytokine TNF-α levels in the brain after ICH [173, 174]. Use of C3 deficient mice has demonstrated similar effects: C3 deficient mice had reduced brain edema, HO-1 positive cells, inflammatory cytokines, and microglial infiltration after ICH [175, 176]. The combined effects of complement-mediated phagocytosis in promoting hematoma clearance and cytokine-induced brain injury warrant further investigation in both ICH and IVH.

#### Systemic inflammation

Although this review has focused on inflammatory events initiated by IVH within the brain, it should be noted that systemic inflammatory changes are a growing focus in other forms of stroke, and particularly cerebral ischemia. With ischemia, signals from the damaged brain can lead to activation of the systemic immune system followed by immunosuppression [177]. The latter may make patients more susceptible to infection. There is a need for studies examining the effect of IVH on the systemic immune system.

### Sex and age differences

The impact of aging and sex differences after IVH and in PHH development are vastly understudied. Both aging and sex are known to impact the immune system [178]. When comparing intraventricular hemorrhage outcomes between young (3-month-old) and aged (18-month-old) rats, the latter had persistently greater ventricular volume from day 3 to day 14 after ictus. Aged rats also had greater ependymal damage, periventricular HO-1 expression, and more choroid plexus macrophage activation than young rats [104]. The fact that aged rats had more evidence of tissue damage and activation of inflammatory cells with subsequent worse hydrocephalus indicates age is an important factor in PHH development. In humans, increased age was significantly correlated with increased need for CSF shunting in adults with IVH [179–181].Sex also appears to be important in IVH and PHH. Studies have demonstrated male infants were more likely to develop IVH, especially high grade IVH, compared to female infants [182–184]. On the topic of subsequent shunt requirement, the data are less compelling: in multiple observational studies of infants and adults with IVH, sex did not significantly correlate with increased risk of shunt placement [179, 185, 186]. In a study comparing young male and female rats after intraventricular thrombin injection, female rats had more severe ventriculomegaly, white matter damage, and neutrophil infiltration into the choroid plexus than their male counterparts [83]. Female rats in this study had more evidence of neuroinflammation and therefore greater ventriculomegaly. As more mechanisms of PHH development are investigated, age and sex differences cannot be ignored.

## Limitations

There are a number of limitations to this review. This was not a systematic review and therefore did not strictly adhere to PRISMA guidelines [187]. This review focused primarily on inflammation within the ventricle, choroid plexus and periventricular tissue. A thorough review of leptomeningeal inflammation was not covered.

## Conclusion

IVH is a significant cause of morbidity and mortality in neonates and adults. PHH causes additional damage to the brain. The pathophysiology of PHH is not well understood. Currently, the management of PHH is limited to temporary or permanent CSF diversion. Obstruction of the arachnoid granulations as the sole cause of PHH is going out of favor as the emerging role other important contributors such as lymphatic/glymphatic absorption and neuroinflammation come to light.. Iron-mediated inflammation, Prx2, thrombin, macrophage activation, cytokines, and complement all appear to contribute to subsequent brain injury, edema and ultimately PHH. As understanding improves of inflammation after IVH, exciting new therapeutic targets will be elicited.

## Data Availability

Not applicable.

## Abbreviations

IVH: Intraventricular hemorrhage
PHH: Posthemorrhagic hydrocephalus
GMH: Germinal matrix hemorrhage
SAH: Subarachnoid hemorrhage
ICH: Intracerebral hemorrhage
CSF: Cerebrospinal fluid
Hb: Hemoglobin
prx2: Peroxiredoxin-2
Hp: Haptoglobin
HO: Heme-oxygenase
AQP: Aquaporin
PAR: Protease activated receptor
PPAR-γ: Proliferator-activated receptor gamma
TNF-α: Tumor necrosis factor-α
TGF-β: Transforming growth factor-β
NF-κB: Nuclear Factor kappa-light-chain-enhancer of activated B cells
MAC: Membrane attack complex
EAE: Experimental autoimmune encephalomyelitis
ICAM-1: Intercellular adhesion molecule-1
VCAM-1: Vascular cell adhesion molecule-1
TLR-2: Toll-like receptor 2
NKCC1: Sodium potassium chloride transporter
LCN2: Lipocalin 2
CLEAR: Clot Lysis: Evaluating Accelerated Resolution of Intraventricular Hemorrhage
MPO: Myeloperoxidase
IBA-1: Ionized calcium binding adaptor molecule 1
OX-6: Major histocompatibility complex II expressed by epiplexus cells
SCH79797: 3-N-cyclopropyl-7-[(4-propan-2-ylphenyl)methyl]pyrrolo[3,2-f]quinazoline-1,3-diamine;dihydrochloride
